# Supplementary material for: Evaluation of knowledge and attitudes regarding Alzheimer’s disease and related dementia among medical students in Palestine: A cross-sectional study
Source: PLoS One. 2024 May 17;19(5):e0304012. doi: 10.1371/journal.pone.0304012 (PMC11101103; doi:10.1371/journal.pone.0304012)
Supplement: Attachment — Submitted filename: Comment1.docx [file pone.0304012.s002.docx]

This study aims to assess the knowledge of Alzheimer's disease and related dementia among medical students throughout Palestine, as well as analyze their attitudes towards dementia.

In addition, they sought to compare outcomes between preclinical and clinical students.

The period of time, participants in the study, tools and methods of data collection, exit criteria and the method of analysis are clearly stated.

Here are some tips to improve your study:

**In the abstract** part of the method section, it is better to mention the time period of the research

**In the introduction:**

In line 37: An extra parenthesis has been added. to be removed

Dementia is an acquired disorder that causes cognitive abilities to decline over time, affecting learning and memory, language, executive function, complex attention, perceptual-motor skills, and social cognition ((1,2). Dementia, a major global health concern.............

**Setting and Study Design**

Lines 61 to 73:

It is mentioned that the study was conducted in 5 universities, but the names of more than 5 universities are given?!!!

Determine the number of participants in each university

The study time is mentioned:

The study participants were recruited between 22-08-2023 and 07-11-2023.

The exit criterion is mentioned:

All undergraduate medical students from the medical schools in Palestine were included in the study except the First-year students. The reason behind excluding the first-year

students is that this study was carried out during their first semester of Medical School which

includes no courses that tackle Alzheimer’s disease or dementia. Medical students who did not consent to participate in this study were excluded.

**Data collection instrument**

The study instrument is explained.

Their research tool consists of three parts

One part of the demographic information and the other two parts are composed of two questionnaires whose validity and reliability have already been confirmed.

It is suggested to mention the validity and reliability results of two questionnaires.

It would be better to express the Likert scale score as well, i.e. from 1 to 7

The tools and methods of data analysis are explained correctly

In the **discussion section**, the results of the study have been compared with other studies and the findings of the research have been discussed

Weaknesses and strengths are pointed out

**The conclusion** of the article is not appropriate and needs to be revised

It is better not to mention the obvious conclusion and state the conclusion of the research.

About 61% of the r**esources** are related to the years 2020 and later.

Final comment:

A good effort has been made to conduct the research, and the stages of the research have been well explained, but considering that they have used pre-approved questionnaires, it is not suitable for this journal.

It would have been better if the questionnaires were converted into the language and culture of the country where the study was conducted and its validity and reliability were re-evaluated.

And the following points were mentioned in the text that

Have the participants been able to have a proper understanding of the questions?

Are the questions consistent with the culture of your country?
