# Supplementary material for: Evaluation of knowledge and attitudes regarding Alzheimer’s disease and related dementia among medical students in Palestine: A cross-sectional study
Source: PLoS One. 2024 May 17;19(5):e0304012. doi: 10.1371/journal.pone.0304012 (PMC11101103; doi:10.1371/journal.pone.0304012)
Supplement: Attachment — Submitted filename: renamed_df650.docx [file pone.0304012.s003.docx]

**Manuscript revision**

**Title:** Evaluation of Knowledge and Attitudes Regarding Alzheimer’s Disease and Related Dementia among Medical Students in Palestine: A Cross-Sectional Study.

**1. Summary**

A cross-sectional study was conducted among Palestinian medical students. Assessing knowledge and attitudes about dementia. Dementia Attitudes Scale (DAS) and Alzheimer's Disease Knowledge Scale (ADKS). As measured by the knowledge of dementia by the students, the overall mean score was ADKS was 18.91 out of 30. The mean ADKS score for students' dementia knowledge was 18.91 out of 30. Students' attitude towards dementia was measured by the DAS, with a mean score of 91.68 out of 140. Higher ADKS scores were observed in clinical students compared to pre-clinical students.

**2. Comments**

A good effort has been put into writing this manuscript. The manuscript provides valuable evidence regarding emphasize the necessity for assessing and upgrading the dementia curriculum in Palestinian medical schools, including adding training modules. However, to make the manuscript better, I have some comments as follows:

1. In the first line of the introduction section of the abstract, no need to There are no statistics. Importance. Dementia can be expressed in another way.
2. Considering that in the title written "Evaluation of Knowledge and Attitudes Regarding Alzheimer's disease and Related Dementia among Medical Students" Why are the clinical and pre-clinical phases compared in the results and discussion section?
3. In the "Setting and Study Design" section, 5 universities are written, but the names of 7 universities are written.
4. In line 120, it is written that an English questionnaire was used, Palestinian students are not native speakers and it may be difficult for them to understand the questions. How do you explain this?
5. This conclusion "Medical students in the clinical phase had higher ADKS scores than students in the pre-clinical phase" is clear. Why was it necessary to conduct a study in this field? Because due to the presence of medical students in the clinical phase, they go to internships and hospitals and have contact with patients, so their experience and knowledge It is more than phase students in pre-clinical.
6. Findings should not be repeated in the conclusion section. The conclusion section should be corrected.
