# Supplementary material for: Evaluation of knowledge and attitudes regarding Alzheimer’s disease and related dementia among medical students in Palestine: A cross-sectional study
Source: PLoS One. 2024 May 17;19(5):e0304012. doi: 10.1371/journal.pone.0304012 (PMC11101103; doi:10.1371/journal.pone.0304012)
Supplement: Attachment — Submitted filename: Response to Reviewers.docx [file pone.0304012.s004.docx]

**Response to Reviewers**

Additional Editor Comments:

Considerable effort has been invested in conducting this research, which is commendable. However, the use of an English questionnaire in an Arabic-speaking country is an ambiguous issue. The reviewers suggest that it would have been better to translate the questionnaire into Arabic and evaluate it before use. Please address this issue and resubmit the revised article. Additionally, please address the other points mentioned by the reviewers.

Response: Thank you for your valuable comment. This paragraph has been added under methodology section: “The DAS was used to assess the medical students' attitudes toward Alzheimer’s disease and related dementia (13). The DAS was developed to measure attitudes towards dementia for both college students and direct care workers (13). DAS internal consistency was originally reported using Cronbach's alpha value of 0.83–0.85. The ADKS test-retest reliability coefficient was 0.81 and Internal Consistency Cronbach's alpha was 0.71 (12). **Furthermore, the questionnaire reliability was reevaluated by conducting a pilot study on 30 students and the total Cronbach alpha was 0.77. As a result, the questionnaire's validity and reliability were assured before conducting the study. The questionnaire was in English, as it is the official teaching language in the medical schools in Palestine**.

Reviewers' comments:

Reviewer #1: A good effort has been made to conduct the research, and the stages of the research have been well explained, but considering that they have used pre-approved questionnaires, it is not suitable for this journal.

It would have been better if the questionnaires were converted into the language and culture of the country where the study was conducted and its validity and reliability were re-evaluated.

And the following points were mentioned in the text that

Have the participants been able to have a proper understanding of the questions?

Are the questions consistent with the culture of your country?

Response: Thank you for your valuable comment. This paragraph has been added under methodology section: The DAS was used to assess the medical students' attitudes toward Alzheimer’s disease and related dementia (13). The DAS was developed to measure attitudes towards dementia for both college students and direct care workers (13). DAS internal consistency was originally reported using Cronbach's alpha value of 0.83–0.85. The ADKS test-retest reliability coefficient was 0.81 and Internal Consistency Cronbach's alpha was 0.71 (12). **Furthermore, the questionnaire reliability was reevaluated by conducting a pilot study on 30 students and the total Cronbach alpha was 0.77. As a result, the questionnaire's validity and reliability were assured before conducting the study. The questionnaire was in English, as it is the official teaching language in the medical schools in Palestine.**

Reviewer #2: The authors have provided a relatively clear and interesting manuscript, yet regrettably, it is poorly written. The objective of this research is to assess the understanding and perspective on Alzheimer's disease and related Dementia among medical students in Palestine. However, there are a couple of remarks that the authors should consider.

1- In the abstract of the article, the method section should also specify the sample size, the year of the study, and the analysis method.

Response: thanks for your valuable comment, the method section in the abstract was elaborated as follows “This cross-sectional study was conducted among 393 medical students in Palestine from August 2023 to November 2023. The assessment of knowledge and attitude toward dementia was measured using the Alzheimer's Disease Knowledge Scale (ADKS) and Dementia Attitude Scale (DAS). The data were analyzed using SPSS version 26, and the Mann-Whitney U-test and the Kruskal-Wallis test were used to compare the means between the groups with significance determined at a P value of less than <0.05.”

2- In the abstract of the article, the result part is poorly written. Written conclusions are not drawn from the results.

Response: Thanks for your comment, the results and the conclusion parts of the abstracts were elaborated as follows:

Results: “The overall mean score of the students’ knowledge of dementia measured by the ADKS was 18.91 (±3.32 SD) out of 30. The mean score of the students’ attitude toward dementia measured by the DAS was 91.68 (±3.32 SD). Clinical students had higher ADKS scores than pre-clinical students (p-value < 0.001). No significant differences in the knowledge and attitudes toward dementia were found between males and females. The medical students' knowledge and attitude scores were positively correlated (ρ= 0.227, p-value <0.001). ”

Conclusion: “Palestinian medical student’s knowledge about Alzheimer's disease and dementia is insufficient, with students in the clinical phase showed better understanding than pre-clinical students. The findings highlight a necessity for enhancing the dementia curriculum and conducting further studies to evaluate training's impact on students' knowledge and attitudes.”

3- Explain how the reliability of questionnaires is assessed in your community.

Response: Thank you for your valuable comment. This paragraph has been added under methodology section: The DAS was used to assess the medical students' attitudes toward Alzheimer’s disease and related dementia (13). The DAS was developed to measure attitudes towards dementia for both college students and direct care workers (13). DAS internal consistency was originally reported using Cronbach's alpha value of 0.83–0.85. The ADKS test-retest reliability coefficient was 0.81 and Internal Consistency Cronbach's alpha was 0.71 (12). Furthermore, the questionnaire reliability was reevaluated by conducting a pilot study on 30 students and the total Cronbach alpha was 0.77. As a result, the questionnaire's validity and reliability were assured before conducting the study. The questionnaire was in English, as it is the official teaching language in the medical schools in Palestine.

4- What formula is used to determine the sample size?

Response: Thanks for your comment. The formula used for sample size calculation was:

The formula for determining the sample size (n) and margin of error (E) is expressed as follows: n = N* x / ((N-1) E2 + x), where N represents the total population size. Given a margin of error (E) of 5% and a confidence level of 95%, with a population size (N) of 15000 and a response distribution of 50%, the formula utilizes a z score of 1.96. The minimum recommended sample size was 375.

5- The sampling technique is incorrect and does not match the description of the Setting and Study Design. This method is employed when the units being studied are rare or not readily identifiable.

Response: Thanks for your comment. In fact, we used a convenient sampling technique. The sentence has been corrected to “A convenient sampling technique was used”. The following sentence has been added; “The participants were recruited by sharing the questionnaire using Google form on their study groups, courses, and their university emails”.

6- The names of the mentioned faculties are more than 5.

Response: thanks for your comment. The sentence has been corrected to “The study took place among the Palestinian Medical Students enrolled in the “Medical Doctor” program in the seven medical schools in five provinces in Palestine: An-Najah National University, Al-Azhar University-Gaza, Palestine Polytechnic University, Arab American University, Islamic University of Gaza, Al-Quds University, and Hebron University”

7- It should be specified in the tables which test was used.

Response: Thank you for your comment, it was incorporated into the revised manuscript, we specified in each table the tests that were used.

8- In the discussion section, the values obtained from other studies should be mentioned. For example, in line 269 "level of knowledge concerning these domains in Norway and China", the average knowledge of the risk factors for each country should be mentioned.

Response: Thank you so much for your comment, it was incorporated in the revised manuscript as follows: “On the other hand, the students’ knowledge of the risk factors of dementia (2.74 (45.7%) ±1.28) and caregiving of patients with dementia (2.63(52.6%) ±1.05) were lower than the reported level of knowledge concerning these domains in Norway (4.19 (69.8%) ±1.21, and 3.96 (79.2%) ± 1.01, respectively) and China (4.09 (68%) ± 1.06, and 3.08 (62%) ± 1.08, respectively)”.

9- In line 313: "The number of participants in this study exceeded the required sample size, which allowed us to generalize the data over the Palestinian medical students" Initially, the total population of your community needs to be determined, followed by determining the necessary sample size. while in the study, the mentioned items are unclear and this statement is incorrect.

Response: Thanks for your comment. The minimum recommended sample size was 375; we increased the sample size to 393 to reduce the random error.

10- Numerous findings have not been incorporated into the discussion; hence, the less significant results should either be excluded or included in the discussion.

Response: Thank you for your comment, at the beginning, we didn’t want to prolong the discussion, but your comment is extremely valuable. We briefly incorporated the results that weren’t previously incorporated into the discussion section in the revised manuscript.

11- References ought to be as current as feasible and should adhere to the citation style of the respective journal.

Response: Based on the journal submission guidelines, the Vancouver citation style was applied for all references.

Reviewer #3: The aim of this article was to evaluate knowledge and attitudes about dementia and Alzheimer's disease among medical students. The authors have a relatively good analysis, but it needs to be revised.

Response: The analysis was redone and the reported results were double-checked. The literature was searched to check the appropriateness of the used tests for our objectives.

The abstract of the article is not well written.

Response: Thanks for your comment. The abstract has been changed to the following:

“Introduction: Dementia, a major global health concern, is an acquired disorder that causes a progressive decline in cognitive abilities, affecting learning and memory, language, executive function, complex attention, perceptual-motor skills, and social cognition. Our study aims to evaluate the knowledge and attitudes regarding dementia and Alzheimer's disease among medical students.

Methods: This cross-sectional study was conducted among 393 medical students in Palestine from August 2023 to November 2023. The assessment of knowledge and attitude toward dementia was measured using the Alzheimer's Disease Knowledge Scale (ADKS) and Dementia Attitude Scale (DAS). The data were analyzed using SPSS version 26, and the Mann-Whitney U-test and the Kruskal-Wallis test were used to compare the means between the groups with significance determined at a P value of less than <0.05.

Results: The overall mean score of the students’ knowledge of dementia measured by the ADKS was 18.91 (±3.32 SD) out of 30. The mean score of the students’ attitude toward dementia measured by the DAS was 91.68 (±3.32 SD). Clinical students had higher ADKS scores than pre-clinical students (p-value < 0.001). No significant differences in the knowledge and attitudes toward dementia were found between males and females. The medical students' knowledge and attitude scores were positively correlated (ρ= 0.227, p-value <0.001).

Conclusion: Palestinian medical student’s knowledge about Alzheimer's disease and dementia is insufficient, with students in the clinical phase showed better understanding than pre-clinical students. The findings highlight a necessity for enhancing the dementia curriculum and conducting further studies to evaluate training's impact on students' knowledge and attitudes. “

Line 68: In this section, the authors mentioned "The study took place among the Palestinian Medical Students enrolled in the “Medical Doctor” program in the five medical schools in Palestine", but in the following, 7 cases are mentioned.

Response: Thanks for your comment, this was a written mistake. The sentence has been corrected to “The study took place among the Palestinian Medical Students enrolled in the “Medical Doctor” program in the seven medical schools in five provinces in Palestine: An-Najah National University, Al-Azhar University-Gaza, Palestine Polytechnic University, Arab American University, Islamic University of Gaza, Al-Quds University, and Hebron University”

Line 86: Do the authors have a justification for using the snowball sampling technique? Because using this sampling method is not suitable for this study.

Response: Thanks for your comment. In fact, we used a convenient sampling technique. The sentence has been corrected to “A convenient sampling technique was used”. The following sentence has been added; “The participants were recruited by sharing the questionnaire using Google form on their study groups, courses, and their university emails”.

Line 87 & 88: The method of calculating the sample size is ambiguous and there is no mention of the calculated size.

Response: Thanks for your comment. The formula used for sample size calculation was:

The formula for determining the sample size (n) and margin of error (E) is expressed as follows: n = N* x / ((N-1) E2 + x), where N represents the total population size. Given a margin of error (E) of 5% and a confidence level of 95%, with a population size (N) of 15000 and a response distribution of 50%, the formula utilizes a z score of 1.96. The minimum recommended sample size was 375.

Line 127-130: The p-values related to the normality test should be mentioned.

Response: Thank you so much for your comment, the p-values of the normality test were added to the sentence as follows: “The Kolmogorov-Smirnov test was used to assess the normality of the continuous data, and it revealed that the age (p-value < 0.001), ADKS (p-value < 0.001), the scores of all the ADKS domains (p-value < 0.001), the DAS (p-value < 0.001), the scores of the dementia knowledge domain (p-value < 0.001), and the score of the Social Comfort domain (p-value < 0.001) were not normally distributed. ”

Line 130: It is better to use Kruskal-Wallis test instead of the Kruskal-Wallis analysis of variance by ranks.

Response: Thanks for your comment. Kruskal-Wallis test was used, and the sentence has been changed to “the Mann-Whitney U-test and the Kruskal-Wallis test was used to compare the means between the groups”

Line 136: The significance level of 5% is sufficient, and there is no need for the things stated in the rest of the sentence.

Response: Thanks for your comment. The rest of the sentence has been deleted and changed to “The significance level was set at 5%.”

Table 7: In Table 7, it is better to distinguish between the results of the Mann-Whitney and Kruskal-Wallis tests. Also, the p-value related to the comparison of Pre-Clinical and Clinical should be corrected.

Response: thanks for your comment. The p-values were corrected in Table 7 as requested.

Not all reported results are discussed.

Response: Thank you for your comment, at the beginning, we didn’t want to prolong the discussion, but your comment is extremely valuable. We briefly incorporated the results that weren’t previously incorporated into the discussion section in the revised manuscript.

Reviewer #4: Manuscript revision

Title: Evaluation of Knowledge and Attitudes Regarding Alzheimer’s Disease and Related Dementia among Medical Students in Palestine: A Cross-Sectional Study.

1. Summary

A cross-sectional study was conducted among Palestinian medical students. Assessing knowledge and attitudes about dementia. Dementia Attitudes Scale (DAS) and Alzheimer's Disease Knowledge Scale (ADKS). As measured by the knowledge of dementia by the students, the overall mean score was ADKS was 18.91 out of 30. The mean ADKS score for students' dementia knowledge was 18.91 out of 30. Students' attitude towards dementia was measured by the DAS, with a mean score of 91.68 out of 140. Higher ADKS scores were observed in clinical students compared to pre-clinical students.

2. Comments

A good effort has been put into writing this manuscript. The manuscript provides valuable evidence regarding emphasize the necessity for assessing and upgrading the dementia curriculum in Palestinian medical schools, including adding training modules. However, to make the manuscript better, I have some comments as follows:

1. In the first line of the introduction section of the abstract, no need to There are no statistics. Importance. Dementia can be expressed in another way.

Response: Thanks for your comment; the introduction section of the abstract was re-written as follows:

Introduction: Dementia, a major global health concern, is an acquired disorder that causes a progressive decline in cognitive abilities, affecting learning and memory, language, executive function, complex attention, perceptual-motor skills, and social cognition. Our study aims to evaluate the knowledge and attitudes regarding dementia and Alzheimer's disease among medical students.

2. Considering that in the title written "Evaluation of Knowledge and Attitudes Regarding Alzheimer's disease and Related Dementia among Medical Students" Why are the clinical and pre-clinical phases compared in the results and discussion section?

Response: In our study, we wanted to evaluate the knowledge and attitudes regarding dementia and Alzheimer's disease among medical students in Palestine, identify the gaps in the knowledge and attitudes, and identify any disparities between the clinical and pre-clinical students to recommend modifications to the dementia curriculum in medical schools in Palestine accordingly. In the results and discussion sections, we compared the level of knowledge and attitudes of medical students in Palestine with the medical students in other countries according to the available studies in Literature, then we dove deep into discussing the differences between clinical and pre-clinical students to have a better understanding of the current level of knowledge and status of medical students’ attitude toward dementia.

3. In the "Setting and Study Design" section, 5 universities are written, but the names of 7 universities are written.

Response: thanks for your comment, this was a written mistake. The sentence has been corrected to “The study took place among the Palestinian Medical Students enrolled in the “Medical Doctor” program in the seven medical schools in five provinces in Palestine: An-Najah National University, Al-Azhar University-Gaza, Palestine Polytechnic University, Arab American University, Islamic University of Gaza, Al-Quds University, and Hebron University”

4. In line 120, it is written that an English questionnaire was used, Palestinian students are not native speakers and it may be difficult for them to understand the questions. How do you explain this?

Response: The DAS was used to assess the medical students' attitudes toward Alzheimer’s disease and related dementia (13). The DAS was developed to measure attitudes towards dementia for both college students and direct care workers (13). DAS internal consistency was originally reported using Cronbach's alpha value of 0.83–0.85. The ADKS test-retest reliability coefficient was 0.81 and Internal Consistency Cronbach's alpha was 0.71 (12). **Furthermore, the questionnaire reliability was reevaluated by conducting a pilot study on 30 students and the total Cronbach alpha was 0.77. As a result, the questionnaire's validity and reliability were assured before conducting the study. The questionnaire was in English, as it is the official teaching language in the medical schools in Palestine.**

5. This conclusion "Medical students in the clinical phase had higher ADKS scores than students in the pre-clinical phase" is clear. Why was it necessary to conduct a study in this field? Because due to the presence of medical students in the clinical phase, they go to internships and hospitals and have contact with patients, so their experience and knowledge It is more than phase students in pre-clinical.

Response: Thank you for your valuable comment, as mentioned above, we wanted to evaluate the knowledge and attitudes regarding dementia and Alzheimer's disease among medical students in Palestine, identify the gaps in the knowledge and attitudes, and identify any disparities between the clinical and pre-clinical students to recommend modifications to the dementia curriculum in medical schools in Palestine accordingly.

Before conducting the study, we expected that clinical students would have a higher level of knowledge compared to pre-clinical students, considering their experience and the nature of their training, yet, we wanted to confirm this hypothesis through our study. We also wanted to identify if there are any differences in the students' attitudes toward dementia and test if it is affected by their phase of training in medical school.

In the results and discussion sections, we compared the level of knowledge and attitudes of medical students in Palestine with the medical students in other countries according to the available studies in Literature, then we dove deep into discussing the differences between clinical and pre-clinical students to have a better understanding of the current level of knowledge and status of medical students’ attitude toward dementia.

6. Findings should not be repeated in the conclusion section. The conclusion section should be corrected.

Response: The conclusion section was re-written as follows:

“Medical students in Palestine have limited knowledge of Alzheimer's disease and related Dementia and a relatively low level of positive attitude toward dementia. The higher the level of students’ knowledge of Alzheimer's disease and related Dementia the more likely they tend to have a positive attitude toward dementia. There is a need for evaluation of the current dementia curriculum in the medical schools in Palestine and a need for the inclusion of more dementia training modules, especially in the pre-clinical phase. Further interventional studies to assess the impact of dementia Training/Education on the knowledge and attitudes of medical students in Palestine are needed.”

Reviewer #5: The study evaluates knowledge and attitudes regarding Alzheimer's disease and dementia among medical students in Palestine using the Alzheimer’s Disease Knowledge Scale (ADKS) and Dementia Attitudes Scale (DAS). The study suggests the need for evaluation of the current dementia curriculum in medical schools and the inclusion of more dementia training modules to improve knowledge and attitudes among all students. Since the findings of this study can be a basis for other future studies, I consider it necessary to pay attention to the following recommendations, which are mentioned in the order of importance for the authors.

1. In this study, the snowball sampling method was used. The snowball sampling method is suitable for research where it is difficult for the researcher to identify people related to the subject. In the present study, it is not difficult to identify medical students to evaluate knowledge and attitudes about Alzheimer's disease and related dementia. Please explain why the authors used this sampling method.

Response: Thanks for your comment. In fact, we used a convenient sampling technique. The sentence has been corrected to “A convenient sampling technique was used”. The following sentence has been added; “The participants were recruited by sharing the questionnaire using Google form on their study groups, courses, and their university emails”.

2. It is mentioned in strengths and limitations: "The number of participants in this study exceeded the required sample size, which permitted us to generalize the data over the Palestinian medical students." It is important to mention that the snowball sampling method is one of the types of non-probability sampling. In non-probability sampling, the sample does not represent the community because more or less samples may be selected from some sections based on the researcher's opinion. Therefore, it seems that the mentioned sentence cannot be scientifically correct.

Response: Thanks for your comment. The sentence was deleted from the paragraph under the strength and limitation section.

3. In section 2.1, states: "The study was conducted among Palestinian medical students enrolled in the "Doctor of Medicine" program at five Palestinian medical schools:", but the seven schools are named in the following sentence.

1. An-Najah National University,

2. Al-Azhar University-Gaza,

3. Palestine Polytechnic University,

4. Arab American University,

5. Islamic University of Gaza,

6. Al-Quds University,

7. Hebron University

Which one is correct? Five or seven?

Response: thanks for your comment, this was a written mistake. The sentence has been corrected to “The study took place among the Palestinian Medical Students enrolled in the “Medical Doctor” program in the seven medical schools in five provinces in Palestine: An-Najah National University, Al-Azhar University-Gaza, Palestine Polytechnic University, Arab American University, Islamic University of Gaza, Al-Quds University, and Hebron University”

4. The authors should include Alzheimer's disease, in addition to dementia, at the end of the introduction on page 4, line 64, as stated in the title.

Response: thanks for your comment. The sentence has been changed as requested as follows “ We aim to evaluate the awareness and attitude of medical students in the State of Palestine regarding Alzheimer’s disease and dementia”

5. In the introduction, line 37, additional parentheses are used to mention the source 1,2.

Response: thanks for your comment. The additional parentheses have been deleted.

6. In Tables 4 and 6, the letter "l" is left in the term "Social Comfort Score".

Response: thanks for your comment. The word has been corrected and written well “social”
