# Supplementary material for: Evaluation of knowledge and attitudes regarding Alzheimer’s disease and related dementia among medical students in Palestine: A cross-sectional study
Source: PLoS One. 2024 May 17;19(5):e0304012. doi: 10.1371/journal.pone.0304012 (PMC11101103; doi:10.1371/journal.pone.0304012)
Supplement: Attachment — Submitted filename: Response to Reviewers.docx [file pone.0304012.s005.docx]

**Response to Reviewers**

Additional Editor Comments:

Journal Requirements:

Please review your reference list to ensure that it is complete and correct. If you have cited papers that have been retracted, please include the rationale for doing so in the manuscript text, or remove these references and replace them with relevant current references. Any changes to the reference list should be mentioned in the rebuttal letter that accompanies your revised manuscript. If you need to cite a retracted article, indicate the article’s retracted status in the References list and also include a citation and full reference for the retraction notice.

Thanks for your comment. All references are according to the journal guidelines. No reference was cited from a retracted article. There are three reference with red color highlighted were changed during previous revision because previously the references were from websites not from articles.

**Additional Editor Comments:**

Thank you for taking into consideration the issues and points raised by the article reviewers. It has been brought to our attention that the reviewers have raised several additional points that require addressing.

Thank you for your feedback. The reviewer’s comments have been addressed as requested.

6. Review Comments to the Author

Reviewer #1: According to the changes made in the article, this article can be suitable with the corrections made.

Response: Thank you for your feedback.

Reviewer #2: Dear Author
Every comment has been addressed accurately. I possess just a single comment.
the abstract of the article, the method section should corrected:
“the Mann-Whitney U-test and the Kruskal-Wallis test were used to compare the mean between the groups with a 5% significance level” Instead “the Mann-Whitney U test and the Kruskal-Wallis test were used to compare the means between the groups with significance determined at a P value of less than”

Response: Thank you for your comment. The sentence has been replaced as requested.

Reviewer #3: A good effort has been put into editing this manuscript. However, to make the manuscript better, I have some comments as follows:

1. Line 154: Considering that your goal is to compare the mean attitude scores in different groups, there is no need to check the normality of the age variable.

Response: Thank you for your comment. The normality of the age variable has been deleted from the statistical analysis section as requested.

2. Table7: The letters a, b related to different tests should be mentioned above the pـvalues.

Response: Thank you for your comment. The letters a, b has been added as requested.
